# Different Temporal Patterns of Specific and General Autobiographical Memories across the Lifespan in Alzheimer's Disease

**DOI:** 10.1155/2015/963460

**Published:** 2015-06-15

**Authors:** Nathalie Philippi, François Rousseau, Vincent Noblet, Anne Botzung, Olivier Després, Benjamin Cretin, Stéphane Kremer, Frédéric Blanc, Liliann Manning

**Affiliations:** ^1^Service of Neurology and CMRR, Neuropsychology Unit, University Hospital of Strasbourg, 1 avenue Molière, 67000 Strasbourg, France; ^2^INSERM, Cognitive Neuropsychology and Pathophysiology of Schizophrenia (U1114), University of Strasbourg, 1 place de l'Hôpital, 12 rue Goethe, 67000 Strasbourg, France; ^3^CNRS, ICube Laboratory (UMR 7357), University of Strasbourg, Pôle API, boulevard S. Brant, Illkirch, 67412 Strasbourg, France; ^4^CNRS, Cognitive and Adaptive Neurosciences Laboratory (UMR 7364), University of Strasbourg, 12 rue Goethe, 67000 Strasbourg, France; ^5^Service of Radiology, Unity of Neuropsychology, University Hospital of Strasbourg, 1 avenue Molière, 67000 Strasbourg, France

## Abstract

We compared specific (i.e., associated with a unique time and space) and general (i.e., extended or repeated events) autobiographical memories (AbM) in Alzheimer's disease (AD). The comparison aims at investigating the relationship between these two components of AbM across the lifespan and the volume of cerebral regions of interest within the temporal lobe. We hypothesized that the ability to elicit specific memories would correlate with hippocampal volume, whereas evoking general memories would be related to lateral temporal lobe. AbM was assessed using the modified Crovitz test in 18 patients with early AD and 18 matched controls. The proportions of total memories—supposed to reflect the ability to produce general memories—and specific memories retrieved were compared between AD patients and controls. Correlations to MRI volumes of temporal cortex were tested. We found different temporal patterns for specific and general memories in AD patients, with (i) relatively spared general memories, according to a temporal gradient that preserved remote memories, predominantly associated with right lateral temporal cortex volume. (ii) Conversely, the retrieval of specific AbMs was impaired for all life periods and correlated with bilateral hippocampal volumes. Our results highlight a shift from an initially episodic to a semantic nature of AbMs during AD, where the abstracted form of memories remains.

## 1. Introduction

Autobiographical memory, which includes both episodic memories and memories belonging to personal semantics, allows a person to remember their personal past recollections and is important for the construction process of personal identity [1]. In its episodic component, autobiographical memory (AbM) refers to personal events that a person is able to reexperience in a unique and detailed spatial and temporal context. This mental time travel requires autonoetic awareness, which consists of the conscious state that accompanies the experience of remembering [2, 3]. For example, remembering in detail a unique event that took place on a specific afternoon in a precise place during one's vacation. Conversely, personal semantic memory (PS; [4]) refers to non-event-based general knowledge about one's life or autobiographical facts and is associated with noetic awareness, that is, the conscious state that accompanies the experience of knowing or familiarity, for example, knowing that one has been on vacation in a general place during a grossly-defined time period, knowing the name or birthday of a relative. Conway and colleagues [1, 5] have also suggested the existence of a third type of qualitatively different autobiographical memories (AbMs), “general” AbMs, consisting of events that are summaries of repeated events or events extended in time or both. For example, the memory of an entire week of vacation or vacation repeated several times in the same place. Aside from their lack of contextual specificity, these memories also differ from specific AbMs by their recollective qualities, being associated with more conceptual and less contextual details. This type of memories constitutes an abstracted version of memories and stand as an intermediate level between personal knowledge and specific AbMs into the hierarchical AbM model described by Conway (the Self Memory System; [6]). In this model, general memories are supposed to be more easily accessible to retrieval and would be the preferred level of entry into episodic AbMs. Additionally to the existence of general AbMs in young and healthy subjects (e.g., [7]), studies of AbM during normal ageing (e.g., [8–10]), emotional disorders [11], and neurological disorders such as Alzheimer's disease (AD; e.g., [12, 13]) have suggested that memories that were initially strictly episodic or “specific” may lose their contextual details and become “semanticized” or “general.” While general memories have only recently been individualized and considered as cognitively distinct from specific memories, some authors tend to assimilate them to PS [14, 15], whereas others clearly contrast them from such personal knowledge that is not confined to a particular time or place [16] or even analyze them using the same phenomenological approach than for specific AbMs [17]. As opposed to the traditional episodic/semantic dichotomy [2], general memories illustrate the overlap between the two components as far as such memories can be considered as an intermediate state on a contextual continuum between specific contextualized and semantic decontextualized memories [18–20]. Concordant with this view, an interdependence between sematic and episodic components was emphasized, namely, regarding AbM, specific memories influence the retrieval of semantic knowledge [21], while general memories constitute the basis from which complex and detailed specific memories are constructed and retrieved [5]. Moreover, semantic knowledge stands as an integral component of specific AbMs, as well as contextual details [1, 10, 22].

Some case studies on retrograde amnesia (e.g., [23, 24]) have suggested that the episodic and semantic components of autobiographical memory have separate neural substrates within the temporal lobes, namely, the lateral temporal neocortex for PS and the medial temporal lobe (MTL) for episodic AbM (review in [25]). Similarly, neurodegenerative diseases involving different lesion patterns in the temporal lobe such as AD and semantic dementia were compared in terms of performances in AbM and PS, confirming different patterns of deficit (e.g., [13, 26–29]), namely, relatively preserved performance in PS for the remote periods in AD and in episodic AbM for the recent period in SD. Some studies of AbM have been conducted over the lifespan, showing a temporal gradient with greater impairment for recent events in AD (e.g., [4, 30–32]). Moreover, the qualitative analysis of remote episodic AbM indicated a loss of contextual details with a pattern of altered specific and preserved general AbMs, accompanied by a shift from autonoetic to noetic awareness, both in AD [12, 13, 33, 34] and amnesic mild cognitive impairment [35, 36]. When taking into account specific memories alone, several studies showed that deficit was ungraded in AD, suggesting that the gradient observed in previous studies was due to the production of general memories [12, 13, 27, 34, 37]. Since AD pathological processes are typically localized in the MTL and to a lesser extend in the lateral temporal neocortex during the early stages of the disease [38], these results suggest that the retrieval of specific memories relies on the MTL no matter how old the AbMs are. Gilboa et al. [39] carried out a neuroimaging study using volumetric analysis and confirmed the anatomical dissociation between AbM and PS within the temporal lobes and documented the dependence of AbM on the MTL region independent of the retention interval. More recently, we confirmed these results with regard to the hippocampal involvement, analyzing the raw scores on the Modified Crovitz Test (MCT) in a voxel-based morphometry (VBM) study [40]. These observations would rather support the multiple trace theory (MTT; [41, 42]) than the standard consolidation theory (SCT, [43]). According to the SCT, declarative memory representations within the neocortex would become stable and independent from MTL structures. Conversely, the MTT suggests that it is the case only for semantic memories, whereas episodic memory retrieval would depend on the MTL for the duration of their existence. The more recent “transformation” theory [44, 45] contrasts the existence of specific and general memories, suggesting that specific memories remain dependent on the hippocampus and that a schematic version with few contextual details would develop in the neocortex as time progresses. Comparing the two categories of memories in a population presenting with MTL lesion could provide the clinical setting to test whether general AbMs are supported by the hippocampus to the same extent as specific AbMs or by other anatomical substrates. Such a study was conducted by St-Laurent et al. [17] in a population of patients with temporal lobe epilepsy, using the Autobiographical Interview [10]. In those patients, the two categories of memories were similarly impaired in terms of “internal” details, suggesting that the recollection of sensory perceptual aspects, but not temporal, specificity relies on the MTL. By contrast, the story elements of the episodes and gist information were preserved, suggesting that this component relies on extra-MTL structures, namely, the lateral temporal neocortex.

The anatomical substrates of the different components of memories have been inconsistently explored with functional imaging studies in healthy subjects. Specific AbM has been extensively explored and associated with a complex neural network [22, 46] including the medial and ventrolateral prefrontal, medial and lateral temporal cortices, the temporoparietal junction, and retrosplenial/posterior cingulate cortices. Among the few fMRI studies which have focused on PS, Maguire and colleagues [47, 48] showed a greater activation of the medial prefrontal cortex, retrosplenial cortex, temporal pole, and temporoparietal junction when contrasting personal with general knowledge. Nevertheless, PS and general semantic memory's activations overlapped in lateral temporal and medial prefrontal regions, suggesting that PS could be considered as a subdomain of semantic memory (review in [20]). Conversely, the few fMRI studies focusing on general AbMs [16, 48–51] showed that they differed from PS insofar they engaged MTL activity. These studies [16, 48–51] suggested a similarity between general and specific AbMs, highlighting a shared and widely distributed network, likely due to perceptual details and contextual information entailed in both categories of memories in healthy subjects (review in [20]). It is worth noting that the activity of some cerebral regions would more particularly depend on the level of specificity. For instance, in some studies [16, 48–51], the medial prefrontal cortex, the parietal cortex (precuneus), and MTL were engaged to a greater extent for the retrieval of specific than general AbMs (review in [20]). Regarding the MTL, Holland et al. [16] suggested that the difference between the two networks depends on the phase of memory formation, with the prefrontal cortex and the MTL being particularly engaged during the construction phase of specific memories. Moreover, the different involvement was related in this study to the specificity of the memories in terms of spatiotemporal context rather than on the richness of details. Conversely, Addis et al. [49] also found hippocampal involvement during the retrieval of general events, which was related to the richness of episodic detail but not to temporal specificity. Regarding the lateral temporal neocortex, activations are usually found in both conditions, notably because semantic representations permeate AbMs, even in their specific form [22]. However, the lateral temporal neocortex is globally involved to a greater extent during the retrieval of general* versus* specific memories, likely reflecting greater engagement of semantic processing for general AbMs [16, 48, 49]. In the same vein, older adults, who produce a higher proportion of general memories, engage the lateral temporal neocortex more than younger adults during episodic AbM retrieval [47]. Taken together, the results of studies performed in healthy subjects tend to contrast general memories and PS and associate the former with the episodic component of AbM [20]. Nevertheless, general memories in healthy subjects differ from those produced by patients with MTL lesion and it might also be the case for their anatomical substrates. Indeed, in healthy subjects, general memories usually consist of repeated events that are associated with perceptual and contextual information, a process that is flawed in patients with MTL lesion [12, 13, 17, 27, 34, 37]. Thus, a correlational approach in patients with neurodegenerative disease such as AD may capture the core anatomical substrates of the abstracted form of memories that may still exist during the early stages of the disease. To our knowledge, no neuroimaging study has yet tackled comparisons between specific and general AbMs in AD, whereas the general component might be critical in AD. General memories not only stand as a transition between episodic autonoetic and semantic noetic memories, they might also support personal identity during the progression of the disease [30].

In the present study, we carried out an investigation on AbM over the lifespan in patients with early AD, comparing them with healthy elderly people, using the Modified Crovitz Test (MCT; [26]). We also investigated the neuroanatomical correlates of the two components of AbM by means of a volumetric analysis of temporal regions of interest. The aims of the present study were (i) to compare the distribution of specific* versus* general AbMs across the lifespan in AD patients compared with healthy elderly controls and (ii) to study the relationship between the specific and general components of AbM and regional volumes of the hippocampus and the lateral temporal neocortex. Tracking the anatomical substrates of general* versus* specific memories among two regions of the temporal lobe classically associated with episodic and semantic memory, respectively, we wished to explore whether the abstracted forms of AbMs are rather associated with the semantic or episodic components of memory. Given that general memories are “semanticized” due to the loss of episodic detail in AD [12, 13, 33, 34], we were expecting a similar pattern of performances than that obtained for PS, that is, relatively spared in the early stages of AD and temporally graded [27, 39]. Moreover, we hypothesized that general AbMs would be supported by anatomical substrates shared with PS, namely, that the ability to provide general AbMs would be related to lateral temporal lobe volume [39]. Conversely, we hypothesized that specific AbMs would be deficient over the lifespan and correlated with hippocampal volume [39, 40].

## 2. Materials and Methods

### 2.1. Participants

Eighteen French AD patients, aged 67 to 85 years, were recruited through the Neuropsychology Unit in the Service of Neurology at the University Hospital of Strasbourg. A diagnosis of probable AD was made according to the criteria of the NINCDS-ADRDA [52] and more recent criteria [53–55]. Each patient disclosed a history of progressive cognitive decline and showed objective impairment of episodic memory with no cueing-related improvement (Free and Cued Selective Reminding Test, [56]), either isolated or associated with other cognitive changes (visuospatial abilities, praxia, language, etc.). All patients had evidence of MTL atrophy [57], using MRI and assessed visually by a senior neurologist (FB). A subset of twelve underwent SPECT examination, which revealed a typical pattern of reduced perfusion in the MTL and parietal regions. Eleven patients underwent cerebrospinal fluid (CSF) biomarkers testing and displayed abnormal results with a combination of low amyloïdAbeta1–42 concentrations (<500 ng/L), increased total tau concentrations (>500 ng/L), and increased phospho-tau concentrations (>60 ng/L). We excluded patients who disclosed a history or symptoms of major depression (Geriatric Depression Scale's score > 6, [58]), cerebrovascular disease, abnormal physical neurological examination, or any other possible causes of dementia. All patients were being treated with one or two AD-specific drugs (acetylcholine esterase inhibitor or acetylcholine esterase inhibitor and memantine). The stage of the disease was mild cognitive impairment (MCI) due to AD or “prodromal” AD in 9 patients, according to the criteria proposed by Albert et al. [55] or Dubois et al. [53], with preserved social functioning and instrumental activities of daily living, that is, with a Clinical Dementia Rating score of 0.5 (CDR, [59]). All these patients had CSF analysis confirming the existence of AD pathology and evolve into a typical AD dementia during their follow-up. The 9 remaining patients had mild dementia with a CDR score of 1. The patients' mean score on the MMSE was 24.3 (SD 2.8). A subset of 15 patients (8 with MCI due to AD and 7 with mild stage of AD) underwent a high resolution MRI scan (3 Tesla), within six months from neuropsychological testing.

Eighteen healthy elderly subjects matched for gender, age distribution, education level, and handedness (see Table 1 for demographic details of the two groups and statistical comparison) were also tested. For inclusion, subjects had to be free of depression (GDS score > 6), central neurological disease, cognitive complaints, or restriction of daily activities (CDR = 0) and without abnormal neurological examinations.

### 2.2. Cognitive Assessment

#### 2.2.1. Anterograde Memory Assessment

Among a larger cognitive assessment, anterograde memory was measured using the Verbal Paired Associates test [60]. With exception of one patient, whose hypoacusia prevented the auditory assessment of the test, patients had a mean score of 9.0 (SD 4.2). Control subjects had normal performances, with a mean score of 18 (SD 1.7).

#### 2.2.2. Specific AbM Task

A French version [61] of the MCT [26] was used to assess AbM. Participants were asked to produce detailed and specific recollections in response to 6 specific cues (e.g., letter, train, surprise, etc.), each prompted five times in order to elicit memories from five periods of life (“0–9 years,” “10–29 years,” “30–59 years,” 60 to current age minus 1 year—referred to as “after 60 years”—and “previous year”; e.g., “Could you recall an event that happened in relation to* a train* before* you turned 9*”). Presentation of the words and time periods were randomized and no time-limit was set. A second cue was given for each possible response before considering that a participant had no memory. The cue was provided as a mean of helping the patient to elicit a memory, and memories not related to the cue word were also taken into account. Similarly, when the memory did not match with the proposed period, it was taken into account for the corresponding period until a number of six memories were obtained for a given period of time. The participants were encouraged to recall as many details as possible about the spatiotemporal context, perceptual details and their emotional state, and incomplete responses were probed with further questions (e.g., “Tell me more.”; “Do you remember where and when it took place?”; “Do you remember other details, e.g., visual details?”; “what did you think or feel ?”). Memories were subsequently scored on a 5-point specificity scale and were divided into five different categories depending: 0, absence of response; 1, semantic facts related to the target word; 2, poorly detailed generic or repeated events; 3, detailed generic or repeated events; 4, poorly detailed specific events; 5, richly detailed specific events. Only memories of events associated with a unique spatial and temporal context were considered as episodic (“specific”) and scored 4 or 5 depending on their level of perceptual details, while other memories were considered “general” (see examples in Table 2). Thirty memories per participant were thus prompted for recollection, with a maximum possible score of 150. Half of these memories were scored by two different raters (NP and LM) with an interrater agreement coefficient of 0.84 using the Krippendorf's alpha test. For the patients the complete assessment was administered in one or two sessions, depending on the patient's fatigability. When necessary, breaks were offered to avoid fatigue. For all patients except two, a relative was present during the autobiographical assessment, allowing the examiner to check for accuracy of the memories.

### 2.3. Temporal Lobe Volumetry Study

A subgroup of 15 patients underwent a high resolution MRI scan, within six months of neuropsychological testing. High resolution anatomical images were obtained with a General Electric SIGNA HDx MR 3T MRI (Milwaukee, USA) using a Fast Spoiled Gradient Echo sequence (TR = 7.2 ms, TE = 2.3 ms, flip angle = 20°, FOV = 22 cm, matrix = 256 × 256, 176 slices of 1 mm).

**Figure 1:**
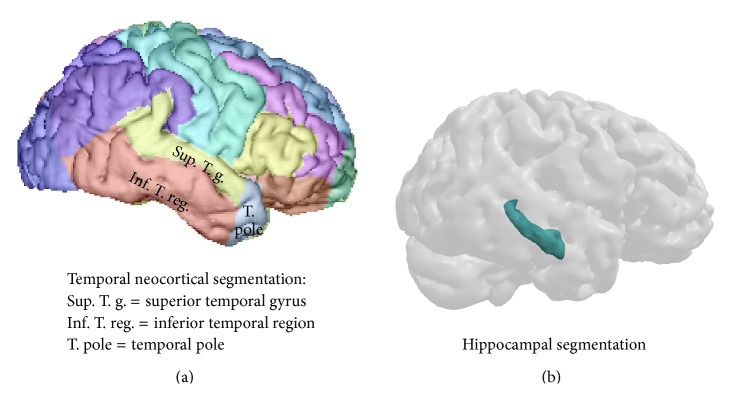
Regional segmentation for the temporal cortex (a) and for the hippocampus (b).

Lateral temporal cortex volumes were extracted in the left and the right hemisphere according to the following subregions: superior temporal gyrus, inferior temporal region—including middle and inferior temporal gyrus—and temporal pole (see Figure 1(a)). This cortical parcellation was done for each patient using a multiatlas label propagation approach. The image processing pipeline consisted of the following steps: (1) a skull-stripping step [62] was performed with the Freesurfer image analysis suite, freely available for download online (http://surfer.nmr.mgh.harvard.edu); (2) each MR image was given B0 nonuniformity correction (using Freesurfer) and ITK-based histogram matching using an arbitrarily chosen patient image as a reference histogram; (3) a set of 16 anatomically annotated images (obtained from the NIREP project, http://www.nirep.org) was registered in a nonlinear way on each patient image [63] using the ANTs software (http://www.picsl.upenn.edu/ANTS); (4) cortical labels were fused [64] using the simultaneous truth and performance level estimation (STAPLE); (5) final cortical parcellation was obtained by fusing logical and estimated labels using STAPLE and the gray matter mask obtained with the Freesurfer image analysis suite.

Hippocampal volume, as well as the total gray matter volume (TGM), was investigated using the subcortical labeling method [65] provided in the freesurfer software (http://surfer.nmr.mgh.harvard.edu/; see Figure 1(b)).

### 2.4. Statistical Analysis

Intergroup comparisons were tested using Student's *t*-test for quantitative demographic characteristics (age distribution and education level). With regard to the MCT, which was primarily designed to elicit specific memories, we broke up the participants' responses into two categories: “specific responses” (i.e., memories scored 4 and 5) and “total responses” (i.e., memories scored 2 to 5), the latter reflecting the ability to elicit “general responses.” This was decided owing to the type of neuroimaging analysis, which was a correlational analysis between volumetric and behavioral data, not a functional analysis. With such a correlational design, the number of “general responses” instead of “total responses” would not reflect meaningful clinical data concerning the ability to produce general responses. Indeed, the milder AD patients would produce more specific and consequently less general memories than patients with a more pronounced deficit, even though they would likely be able to elicit a general version (i.e., less detailed) of the specific memories, according to the previously described pattern of altered AbM in AD (See paragraph 2 of the introduction). For instance, a patient able to produce 30 “total responses” including 15 “specific responses” and 15 “general responses” would have been able to produce 30 “general memories”. Conversely, as general memories are produced consecutively to the lack of specific responses, AD patients with more pronounced deficit would produce a higher proportion of general memories. For instance, a patient producing 21 “total responses” including only 2 “specific responses” and 19 “general responses” would have a higher number of “general responses” than the milder patient, whereas his true ability to produce general memories would be inferior. Therefore, the “total responses” category rather than the “general responses” is suited for correlational analyses. The results were expressed in terms of “rates” in percent, relative to the number of possible responses (scored 0 to 5) for the two categories, namely, 6 per participant for each period of life and 30 over the entire lifespan. ANOVAs were performed in order to compare mean rates of responses obtained in these two categories, as a function of the different periods of life, both within and between the two population groups. In order to control for possible violations of the homogeneity of variance associated with analysis of binary data summarized as percentages, an arcsine transformation was performed on the rates of memories and statistical tests were conducted using the transformed data. After transformation of data, normality of the distribution was verified (Kolmogorov-Smirnov test) and post hoc analyses were performed (Newman-Keuls test).

To investigate the relationships between AbM performance and anterograde memory in AD patients, we performed analyses using Pearson's correlation test between rates of total or specific responses produced during the MCT and scores on the Verbal Paired Associates. Correlations were tested over the global lifespan then independently for each period of life.

Correlation analyses between the different brain volumes and behavioral data were carried out using a general linear model with age and total gray matter volume (TGM) as nuisance covariates with the “statsmodels python module” (http://statsmodels.sourceforge.net). Corrections for multiple analyses were performed when testing the different periods of life or the different lateral temporal subregions, using a statistical threshold of *P* = 0.01.

## 3. Results

### 3.1. AbM Performances on the MCT

**Figure 2:**
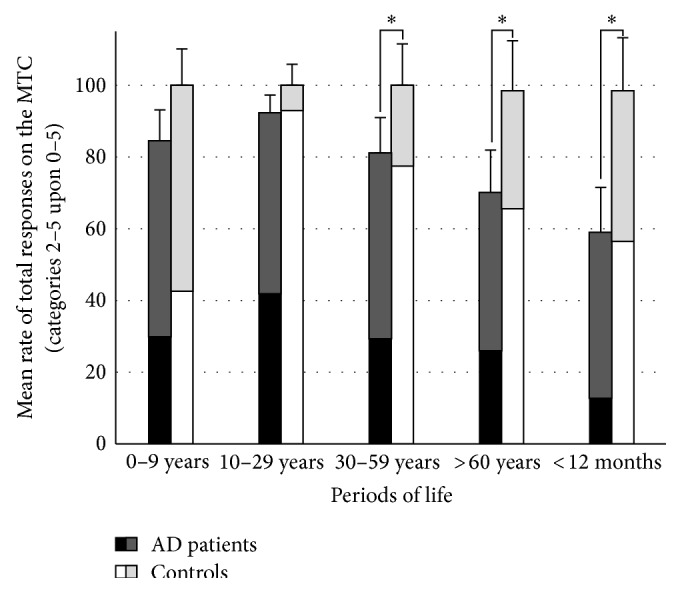
Mean and standard deviation for the rates of total responses (number of memories scored (2 to 5)/30) on the MCT for AD patients and healthy controls in the five periods of life (^*^significant intergroup comparison after post hoc analysis). The rates of specific (scored 4 and 5) and general responses (scored 2 and 3) among the total responses are indicated in black and dark gray for the AD patients, and in white and light gray for the controls, respectively.

**Figure 3:**
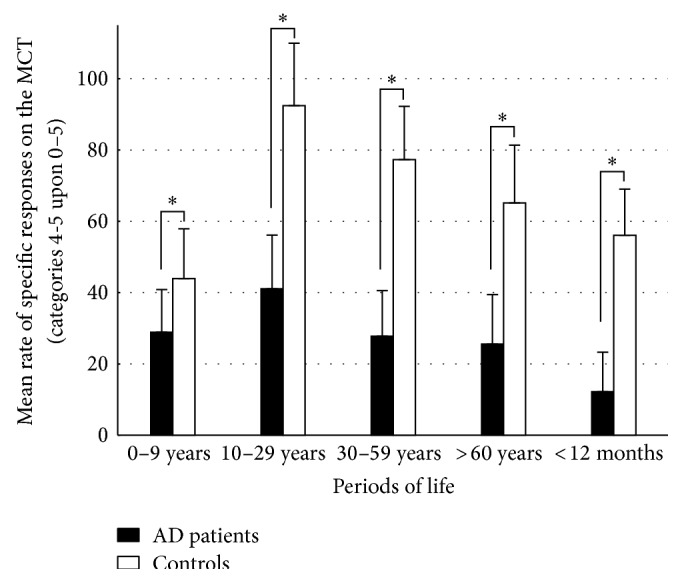
Mean and standard deviation for the rates of specific responses (number of memories scored (4 and 5)/30) on the MCT for AD patients and healthy controls in the five periods of life (^*^significant intergroup comparison after post hoc analysis).

For both groups, mean rates of responses on the MCT obtained for each category are shown for the five periods of life (“total responses” and “specific responses” are shown in Figures 2 and 3, resp.).

#### 3.1.1. Intergroup Comparisons on the MCT

*Rates of Total Responses (Memories Scored 2 to 5/30).* Statistical analyses revealed that AD patients gave significantly fewer total responses over the lifespan than controls in response to the MCT cue-words (*F*_[1,34]_ = 30.22; *P* < 0.001). ANOVAs showed a significant period effect (*F*_[4,136]_ = 11.89; *P* < 0.001) and a significant group × period interaction (*F*_[4,136]_ = 4.9; *P* < 0.001). Post hoc analysis indicated that AD patients exhibited significantly fewer total responses than controls for the three more recent periods of life (*P* < 0.001 for the “previous year” and “after 60 years” periods and *P* = 0.005 for the “30–59 years” period). Other comparisons did not reveal significant differences between the groups.

*Rates of Specific Responses (Memories Scored 4-5/30).* AD patients recollected significantly fewer specific responses than controls (*F*_[1,34]_ = 49.34; *P* < 0.001). ANOVAs showed a significant period effect (*F*_[4,136]_ = 14.51; *P* < 0.001) and a significant group × period interaction (*F*_[4,136]_ = 2.38; *P* = 0.05). Post hoc analysis revealed that AD patients performed worse than controls over the whole lifespan (*P* = 0.03 for the “0–9 years” period, *P* < 0.001 for the “10–29 years,” “30–59 years,” and “after 60 years” periods and for the “previous year”).

#### 3.1.2. Interperiod within Group Comparisons on the MCT

*In the Healthy Control Group.* Post hoc analysis revealed no significant differences when comparing mean rates of* total responses* between one period and another. However, there were significantly fewer specific responses for the “0–9 years” period than for the “10–29 years” (*P* < 0.001), and “30–59 years” periods (*P* = 0.004). The mean rate of* specific responses* was significantly higher in the “10–29 years” period compared with the remaining periods (*P* < 0.001 for the “0–9 years” and “previous year” periods, and *P* = 0.002 for the period “after 60 years”) except for the period “30–59 years” (*P* = 0.68). Other comparisons did not reveal significant differences between mean rates.

*In the AD Group.* Post hoc analysis revealed that mean rates of* total responses* were significantly inferior for the “previous year” period as compared with the three first periods (i.e., the “0–9 years,” “10–29 years,” and “30–59 years” periods, *P* < 0.001 in every case). It was also the case for the period “after 60 years” compared with the two first periods (*P* = 0.005 for the “0–9 years” period and *P* < 0.001 for the “10–29 years” period). The mean rate of* specific responses* was significantly higher in the “10–29 years” period as compared with the “previous year” only (*P* < 0.001). Other comparisons did not reveal significant differences between mean rates.

### 3.2. Correlation Analyses between AbM and Anterograde Memory Performance

Correlations between the two AbM category scores and scores on anterograde memory (Verbal Paired Associates) in the AD group were tested (shown in Table 3). A significant correlation was obtained between the rates of responses on the MCT over the lifespan and scores on the Verbal Paired Associates test for the category of total responses (*N* = 18; *R* = 0.59; *P* = 0.01) and for the category of specific responses (*N* = 18; *R* = 0.71; *P* = 0.001). Interestingly, correlations also existed between the scores on the Verbal Paired Associates and the rates of specific responses independently for the “10-29 years” period (*N* = 18; *R* = 0.64; *P* = 0.006), “30–59 years” period (*N* = 18; *R* = 0.39; *P* = 0.007), and “after 60 years” period (*N* = 18; *R* = 0.67; *P* = 0.003). It was also the case with the rate of total response for the “previous year” (*N* = 18; *R* = 0.59; *P* = 0.01) and the “after 60 years” period (*N* = 18; *R* = 0.49; *P* = 0.04). This last correlation did not remain significant after correction for multiple analysis due to the five periods of life, using a threshold of *P* = 0.01.

### 3.3. Correlation Analyses between AbM and Brain Regional Volumetry

**Figure 4:**
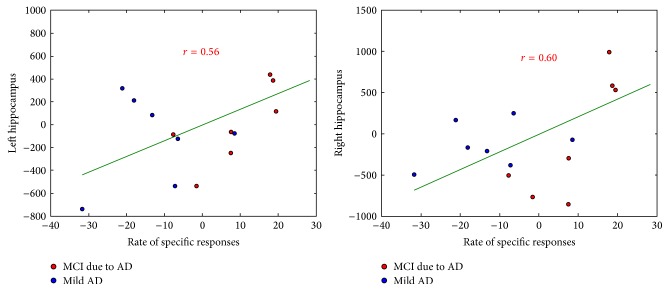
Correlations of the rate of specific responses (number of memories scored (4 and 5)/30) on the MCT with hippocampal volumes, including age and TGM volume as nuisance covariates (red: MCI due to AD, blue: mild AD).

**Figure 5:**
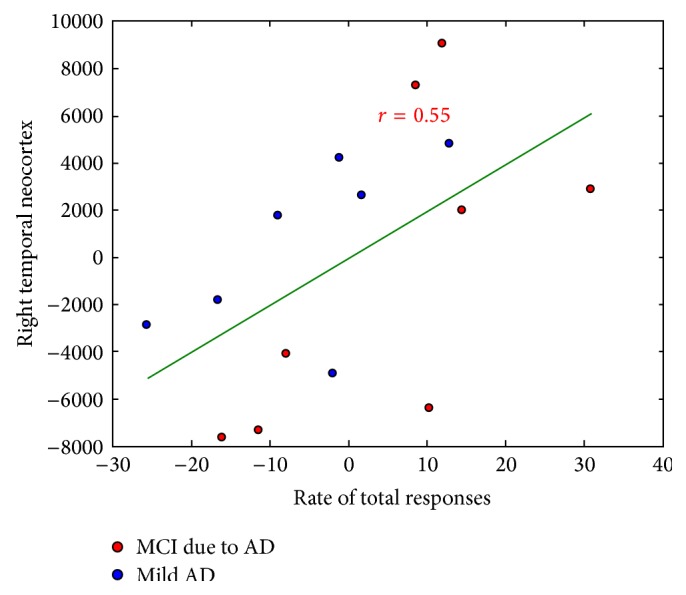
Correlations of the rate of total responses (number of memories scored (4 and 5)/30) on the MCT with the right temporal neocortical volumes, including age and TGM volume as nuisance covariates (red: MCI due to AD, blue: mild AD).

Correlations between the two AbM category scores with hippocampal and lateral temporal neocortex volumes were tested on 15 AD patients, taking age and TGM volume as nuisance covariates.  Table 4 shows the results of the correlation analyses using the global cortical volume of these regions, scatterplots for the significant correlations are shown in Figures 4 and 5.

#### 3.3.1. Correlations with Hippocampal Volume

For scores* over the global lifespan*, there were correlations between the hippocampal volume and the rate of specific responses on the MCT for both sides (*P* = 0.03, *r* = 0.56 on the left; *P* = 0.02, *r* = 0.60 on the right; see Figure 4), whereas it was not the case for the rate of total responses (*P* = 0.20, *r* = 0.28 on the left and *P* = 0.07, *r* = 0.46 on the right).* Independently per period of life,* a significant correlation was also found between the rate of specific responses and the bilateral hippocampal volume for the three intermediate period of life, that is, “10–29 years”, “30–59 years” and “after 60 years” periods (*P* = 0.03 and *r* = 0.53, *P* = 0.02 and *r* = 0.57, *P* = 0.008, and *r* = 0.65, resp. on the left; *P* = 0.02 and *r* = 0.58, *P* = 0.009 and *r* = 0.64, *P* = 0.007, and *r* = 0.66, resp. on the right). Interestingly, the rate of total responses was also correlated with right hippocampal volume for the “previous year” (*P* = 0.03 and *r* = 0.52). Note that only results with *P* ≤ 0.01 should be taken into account after correction for multiple analysis due to the five periods of life. Overall, these results are coherent with those obtained using the scores on the Verbal Paired Associates, since they were correlated with both the left (*P* = 0.001 and *r* = 0.75) and the right (*P* = 0.01 and *r* = 0.63) hippocampal volumes.

#### 3.3.2. Correlations with Lateral Temporal Neocortical Volume

**Figure 6:**
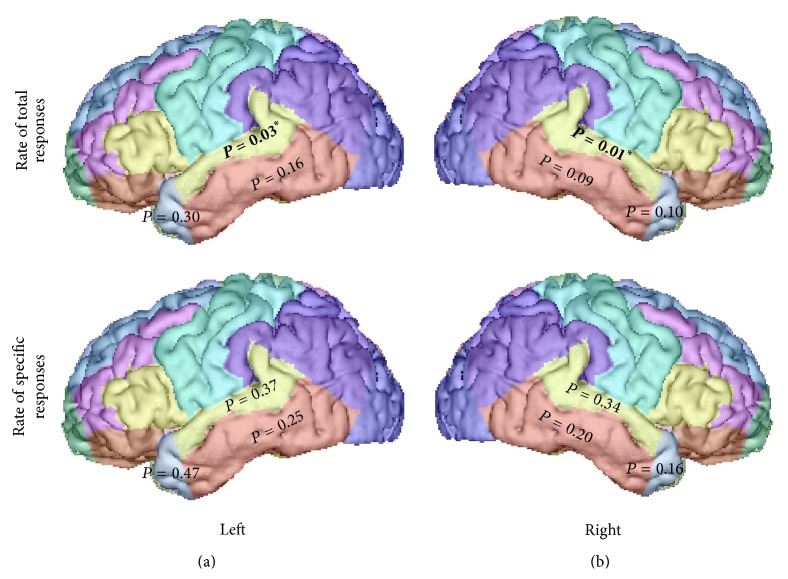
Correlations in AD patients between rates of specific or total responses over the lifespan on the MCT, and the volume of the temporal neocortex subregions (bold and ^*^indicate significant correlations).

For scores* over the global lifespan*, we found correlations between global lateral temporal neocortical volume and the rate of total responses on the MCT for the right side (*P* = 0.03, *r* = 0.55; see Figure 5), but not the left (*P* = 0.12, *r* = 0.37), which was not the case for the rates of specific responses (*P* = 0.17, *r* = 0.30 on the right and *P* = 0.30, *r* = 0.17 on the left). When the analyses where tested* on the basis of the temporal subregions*, as it is shown in Figure 6, we found a significant correlation between the rates of total responses on the MCT and the volume of the superior temporal gyrus on the left (*P* = 0.03, *r* = 0.54) and on the right (*P* = 0.01, *r* = 0.62). Only the correlation obtained with the right superior temporal region would remain significant after correction for multiple analysis due to the presence of three subregions on each side.

## 4. Discussion

In the present study, we sought to investigate the distinct pattern of specific and general (reflected by the rate of “total responses”) AbMs deficit in AD and the underlying neuroanatomical correlates within the temporal lobes. We have documented (i) impaired total AbMs following a temporal gradient which spares remote memories, resulting from preserved remote general memories, while retrieval of specific AbMs was impaired for all life periods; (ii) a correlation between total AbMs and the volume of the right lateral temporal neocortex (superior temporal gyrus), which suggests that this structure plays a key role in the ability to provide general memories in AD; (iii) correlations between specific AbMs and anterograde memory scores, and bilateral hippocampal volume.

Regarding total AbMs across the lifespan in AD (see Figure 2), a temporal gradient appears with more impaired memories seen with shorter retention intervals, in agreement with the majority of studies on AbM in AD patients [4, 13, 26, 30–34, 66–68]. The better performances in AbM for the most remote periods relative to the recent one, result from the production of “general” memories, compensating the lack of specific AbMs, whose pattern of deficit appeared ungraded in the AD patients (see Figure 3 and paragraph 3 of the discussion). Overall, the AD patients produce relatively more general memories than the controls, because memories that were originally episodic have lost their contextual specificity and their level of episodic detail and have become “general” (e.g., [12, 13]). Nevertheless, the healthy controls cannot be thought as being less capable than the patients to produce general memories, since they would most likely be* able* to produce a general version of their specific AbMs. This would also be the case for AD patients with milder AbM deficit that are still able to produce some specific memories. Therefore, the rate of total responses was considered to reflect the ability to produce general AbMs in our study. Our volumetric correlation analyses indicate that these memories (i.e., the rate of “total responses”) are associated with the lateral temporal neocortex (superior temporal gyrus). The implication of the temporal neocortex for PS (review in [20]) has already been demonstrated [39, 47, 48] and is widely agreed for general semantics (e.g., [69], review in [70]). The temporally graded retrograde amnesia for general memories in our study and for PS in Gilboa et al.'s study [39] likely reflects the progression of AD within the lateral temporal neocortex. The superior temporal gyrus seems particularly involved for general memories in our study, prominently on the right hemisphere, whereas only a trend was found on the left side after correction for multiple analysis. The superior temporal gyrus was included in both anterior and posterior temporal regions defined by Gilboa et al. [39], as being associated with PS bilaterally, prominently on the left side. In fMRI studies conducted in healthy subjects, the activation of the lateral temporal cortex sustaining general memories was also identified by Holland et al. [16] in the left superior and middle temporal gyri (BA 21/22) and by Addis et al. in the right inferior temporal gyrus [49]. While it is difficult to account for the inconsistencies regarding the temporal subregion involved in the different studies and to carry out a direct comparison between general memories and PS or between correlational and functional imaging, our results suggest that general memories share common anatomical substrates with semantic memory.

Regarding the implication of the hippocampus in the retrieval of general AbMs, we found a deficit in total responses for the most recent period of life, which likely results from an additional encoding and/or consolidation deficit secondary to hippocampal dysfunction. Indeed, the anterograde memory deficit, which was previously well-documented with respect to “laboratory” memory in AD (e.g., [71]), would not spare real-life events. This hypothesis was corroborated by the positive correlation between the MCT rate of total responses for the “recent year” period and scores on the Verbal Paired Associates test (review in [72]) and finally by the fact that both scores were correlated with the hippocampal volume. We found no correlation between the rate of total memories and hippocampal volumes, for the remaining life periods or the global lifespan, as opposed to what was found for the rate of specific memories (see paragraph 3 of the discussion). However, hippocampal activity was observed during the retrieval of general events in fMRI studies involving healthy subjects [16, 49]. The qualitative difference in terms of perceptual and contextual details between general memories produced by AD patients and those elicited by healthy subjects might explain the inconsistency in hippocampal implication. Indeed, if general memories are defined as summaries of repeated events or events that extended in time, healthy subjects cannot prevent themselves from remembering a single instance associated with the general event during an fMRI task [20]. Moreover, general events in healthy subjects entail perceptual details, the level of which was related to hippocampal engagement in Addis et al.'s study [49], rather than the temporal specificity of the memory. Conversely in AD patients, from AbMs that were initially episodic only remains a framework of semantic knowledge devoid of perceptual and contextual detail [12, 13, 33, 34], which seems supported by extrahippocampal structures such as the lateral temporal neocortex [17]. However, we acknowledge that the MCT is based on a qualitative scoring, and the fact that the perceptual and contextual aspects of the recollection were not quantitatively scored might have masked potential correlations with the hippocampal volumes. Finally, the different nature of functional* versus* correlational analyses might also explain the different hippocampal implication, since fMRI studies reveal finer cerebral activations than correlational analyses. Overall, even though general AbMs share common characteristics (perceptual details, contextual information, first person perspective) with episodic memory [20] and constitute the preferred entry level into specific AbMs [6] in healthy subjects, our results suggest that,* in AD patients*, general memories are* preferentially—*rather than* exclusively*—supported by the lateral temporal neocortex, reflecting the semantic nature of the gist of AbMs that is more resilient to the disease.

When taking into account specific memories (scored 4 and 5), an ungraded deficit appears in AD patients as compared with controls, that is, for all the lifespan (see Figure 3). More particularly, healthy controls produced more instances of specific episodic AbMs for the “10–29 years” period than for the rest of the lifespan, which is consistent with the “reminiscence bump” described during normal ageing in previous studies [73]. This effect is interpreted, in the self-narrative theory [73], as a modulation of encoding and storage of “self-defining” event memories from adolescence and early adulthood that are more easily accessible. Importantly, in our study, AD patients did not elicit significantly more specific episodic memories for this young adulthood period relative to other life periods, except for the latest period of life. Within the control group, lower scores were observed for the “0–9 years” period as compared with subsequent periods in the controls. This was previously described during normal ageing as “childhood amnesia” [73] and is thought to reflect the inability to encode information in an episodic modality [3, 74, 75]. This effect also disappeared in the patients since the AbM deficit was observed for all the lifespan. Lastly, impaired encoding and/or consolidation processes account for the lower scores in the most recent period of life, in parallel with what mentioned above for general memories (paragraph 2 of the discussion). Differently stated, the pattern of extended retrograde amnesia and the absence of a reminiscence bump effect in the AD group are compatible with the ungraded AbM retrieval deficit found in previous studies involving AD patients when considering strictly episodic memories solely [12, 13, 27, 37]. Irish et al. [35] found similar results in amnestic mild cognitive impairment, suggesting that this alteration exists at the earliest stages of the disease, even though these patients were not confirmed to be AD patients with amyloid markers. Comparing two different interview methods in AD (the Autobiographical Interview [10] and the Autobiographical Memory Interview [4]), Barnabe et al. [68] demonstrated that the nature of the AbM test impacts the pattern of findings, the temporal gradient being brought out by fewer periods and masked by fewer memories per period. Even though our test entailed a rather large number of periods, which might have favored the absence of a temporal gradient, the fact that six memories per life period were collected should have had the opposite effect. Not only was the deficit in specific AbMs ungraded in our study, but it also correlated with anterograde memory scores, as was the case in previous studies [12, 39], suggesting the implication of the MTL. Moreover, this correlation existed independently for the three intermediate periods in our study. In line with our previous work in VBM [40] and with Gilboa et al.'s [39], we were able to relate the deficit of specific events to bilateral hippocampal atrophy. This was also the case independently for the three intermediate periods, that is, for an extended retention interval, though only at the level of a trend for the “10–29 years” period after correction for multiple analyses. Note that no correlation was found for the first period likely because the deficit of specific AbMs for this period is due to the “childhood amnesia,” rather than to a retrieval deficit explained by atrophy. Conversely, no correlation was found with the lateral temporal neocortex (see paragraph 4 for further discussion). In other words, our study supports the existence of an extended deficit of specific AbMs* prominently* due to hippocampal atrophy within the temporal lobe in AD.

Because direct comparison with fMRI studies in healthy subjects is limited by major methodological differences, we will exert caution concerning the further extrapolation of our results to normal functioning and discussion of the different theories of consolidation. Nevertheless, our results suggest that within the temporal lobe, the retrieval of specific memories would prominently and extensively depend on the hippocampus, while a general version would exist independently in the neocortex. Together with previous correlational studies in AD [39, 40] and with fMRI studies performed in healthy elderly subjects (e.g., [76–78]), these results support the MTT [41, 42], which posits that the retrieval of episodic memories would depend on the MTL for the duration of their existence. Conversely, in the SCT [43], a correlation would have been expected between the deficit of AbMs and the neocortical volume, independent of the category “general” and “specific.” Namely, we found no correlation between the rate of specific memories and the volume of the temporal neocortex, contrary to what was observed for the general memories. Our results seem to be eloquent when considering Winocur et al.'s transformation theory [44, 45]. Indeed, the authors not only postulate an extensive dependence of episodic AbMs retrieval on the hippocampus but additionally implicate the neocortex in the ability to provide a schematic version of recollections. Though, according to this theory, interplay is supposed to exist between the two categories of memories, which we acknowledge was not possible to illustrate in our study given the binary classification of memories. Indeed, rather than considering the two memory systems in isolation, theorists tend to consider that the semantic and episodic components of memory are inter-dependent, namely, with the association of episodic details to general AbMs and the implication of semantic knowledge during the retrieval of specific AbMs [18, 19]. Besides, the temporal neocortex is frequently activated in fMRI studies during the retrieval of specific events, likely reflecting the semantic component entailed in AbMs [19, 20, 22]. The fact that we found no correlation between the rate of specific memories and the lateral temporal neocortex might be explained by methodological differences between functional and correlational approaches, as was previously discussed (see paragraph 2 of the discussion). Moreover, given our hypothesis, our study focused only on the temporal lobe, but the neuroanatomical substrates of AbM are more complex, extending beyond the temporal lobe, as revealed by fMRI studies in healthy subjects [9, 22]. Therefore, other neocortical regions also affected by AD would likely be involved when analyzing the whole brain, such as the posterior cingulate cortex [79], the retrosplenial cortex, the precuneus, the anterior cingulate cortex, the ventromedial prefrontal cortex [40]. Overall, our findings should not be considered as supporting the* exclusive* but rather the* preferential *implication of the hippocampus for specific AbMs within the temporal lobe, similarly to the implication of the lateral temporal neocortex for general AbMs (see paragraph 2 of the discussion). Whether general memories stand as a transition step on a continuum between episodic and semantic memory [18–20], they might be closer to the episodic end of the continuum in healthy subjects. This suggestion is based on common characteristics such as perceptual and contextual information [20] and the fact that they constitute the preferred entry level into specific AbMs [6]. During the early stages of AD, general memories would undergo a shift towards the semantic end of the continuum.

The present study has several limitations. Firstly, our sample size was relatively small, preventing stratification into two sub-groups (“MCI due to AD” and “mild AD”) and revealing some correlations only at the level of a trend. Secondly, because we carried out a correlation approach, we endeavored to study the ability to produce general memories by using the rate of total responses, whereas an fMRI study would have allowed observing the cerebral regions directly associated with the retrieval of general memories. Additionally, the fact that we used a region of interest analysis has limited our findings to the temporal lobe, whereas other regions also affected by AD would have likely been involved. A further limitation is that the MCT was not originally conceived for the assessment of general memories [16], which then stand as a substitute for the lack of episodic memories. Finally, a more precise scoring method including a structured specific probing could have allowed distinguishing between different qualitative aspects of the recollection such as the specificity in time and place* versus* number of episodic details recollected. In particular, constraining our analysis to the rate of memories classified in each category rather than to quantitatively scoring the episodic and semantic information included in the memories (e.g., “internal” and “external” details with the Autobiographical interview [10]) prevented us from demonstrating the existence of a subtler transition from one category to another in AD.

## 5. Conclusion

In the present study, we have characterized AbM deficit during the early stages of AD (i) on the basis of a reliable number of recollections, (ii) in patients whose diagnosis was based on stringent criteria [53–55], (iii) distinguishing between specific and general (i.e., “total”) AbMs, (iv) with regard to cerebral volume in regions of interest. We were able to confirm different temporal patterns of AbM deficits in AD: specific AbMs were impaired for an extended retention interval secondary to hippocampal deficit, whereas preserved general (i.e., “total”) AbMs followed a temporal gradient sparing remote periods of life and relying on the temporal neocortex. Our results suggest a shift from an initially episodic to a semantic nature of AbMs during early AD, where the remains of memories consist of a framework of knowledge devoid of episodic detail.

## Figures and Tables

**Table 1:** Comparison of the general data for the two groups (F: female; M: male; L: left; R: right; SD: standard deviation).

	*N*	Sex ratio (F/M)	Mean age (SD) in years	Mean education level (SD) in years	Hand dominance ratio (L/R)
Control group	18	4/14	73.67 (5.37)	13.17 (3.01)	1/17
Patient group	18	4/14	77.17 (6.42)	12.17 (6.68)	1/17
Statistical analysis	—	—	*t*[34] = 1.17 *P* = 0.08	*t*[34] = −1.05 *P* = 0.3	—

**Table 2:** Examples of scoring on the MCT.

MCT score	Contextual specificity	Examples (*Cue-word proposed*)
(5) Richly detailed specific events	+	*Boat*: I was in Alger for professional reasons, and I had to take the plane to go to Casablanca. In the waiting room, I was intrigued because a lot of people spoke Russian. I asked repeatedly the policemen to confirm that the plane was really going to Casablanca. I finally boarded the plane, it was an Illyushin. Once seated in the cabin, my neighbor, who was an Algerian, finally revealed to me that the plane was actually going to Moscow! I was afraid of not being able to go out of the plane anymore and to leave for Russia… but I finally managed to leave the cabin and to join the right flight … I was around 58, it must have been in September, it was still very warm … I was there on my own.

(4) Poorly detailed specific events	+	*Damaged*: At the age of 10, one day of September, 1943, Strasbourg was bombarded by the Germans. The weather was warm and clear. My parents' house was next to the river Ill. Suddenly, we saw bombs falling everywhere… “What happens?” Neudorf had already been bombarded and we understood rapidly what was currently going on. Houses all around us collapsed. We went to take refuge in the basement. I was scared… I was with my parents and there were also neighbors, it seem to remember.

(3) Detailed generic or repeated events	−	*Train*: When I was 9, the family acquired a black Citroën. Since then, we used to go regularly to the valley of X to picnic. My father usually set off to pick mushrooms and stayed for hours… My mother had to call him back shouting so that he could hear her and return. Meanwhile, my sister, my mother and I used to pick blackberries. My mother used to cooked blackberries in all possible and unimaginable ways… Now, I cannot eat them anymore.

(2) Poorly detailed generic or repeated events	−	*Happy*: When I was 3 years old, my parents settled down in Strasbourg, and we lived next to a childless couple. The husband was a doctor. His wife, X, liked me very much and often took care of me.

(1) Semantic facts	−	*Letter*: In Alsace at that time, before going to the school, we went to the “Kindergarten”

**Table 3:** Correlations in AD patients between the rates of specific autobiographical events or the rates of total memories on the MCT, and anterograde memory performances on the Verbal Paired Associates, over the lifespan and independently for each period of life (Bold and ∗ indicate significant correlations with *P* < 0.05, and ∗∗ with *P* < 0.01).

Rates of responses on the MCT	Total responses and verbal paired associates (*N* = 18)		Specific episodic events and verbal paired associates (*N* = 18)	
*P*	*R*	*P*	*R*

Over the lifespan	**0.01** ^*^	0.59	**0.001** ^**^
“0–9 years”	0.18	0.34	0.17
“10–29 years”	0.11	0.40	**0.006** ^**^
“30–59 years”	0.27	0.28	**0.007** ^**^
“after 60 years”	**0.04** ^*^	0.49	**0.003** ^**^
“previous year”	**0.01** ^*^	0.59	0.1

**Table 4:** Analysis shows correlations in AD patients between rates of specific autobiographical events or total memories over the lifespan on the MCT and the regional volume of the hippocampus and temporal neocortex, including age and TGM volume as nuisance covariates (Bold and ∗ indicate significant correlations).

Rates of responses on the MCT over the lifespan	Hippocampus (*N* = 15)		Temporal neocx (*N* = 15)	
Left	Right	Left	Right

Total responses	*P* = 0.20	*P* = 0.07	*P* = 0.12
	*r* = 0.28	*r* = 0.46	*r* = 0.37

Specific episodic events	*P* = **0.03** ^*^	*P* = **0.02** ^*^	*P* = 0.30
	*r* = **0.56**	*r* = **0.60**	*r* = 0.17
